# Comprehensive analysis of UBE2C expression and its potential roles and mechanisms in hepatocellular carcinoma

**DOI:** 10.18632/aging.204792

**Published:** 2023-08-14

**Authors:** Xiao Li, Zhaosheng Ma, Linhang Mei

**Affiliations:** 1 Department of Emergency, Taizhou Hospital of Zhejiang Province Affiliated to Wenzhou Medical University, Taizhou, Zhejiang 317000, China; 2 Department of Oncological Surgery, Taizhou Hospital of Zhejiang Province Affiliated to Wenzhou Medical University, Taizhou, Zhejiang 317000, China

**Keywords:** UBE2C, hepatocellular carcinoma, liver cancer, diagnosis, prognosis, microRNA (miRNA)

## Abstract

Hepatocellular carcinoma (HCC) ranks one of the most common and lethal cancers all over the world. Previous studies suggest that ubiquitin-conjugating enzyme E2C (UBE2C) serves as an oncogene in human cancers. However, its expression, diagnosis, prognosis and potential mechanisms in HCC remain largely unknown. In this study, the expression of UBE2C in HCC was first analyzed by comprehensive bioinformatic analysis. ROC curve analysis and survival analysis were employed to assess the diagnostic and prognostic roles of UBE2C in HCC. UBE2C promoter methylation level and upstream regulatory miRNAs of UBE2C in HCC were explored. The present work demonstrated that UBE2C was significantly upregulated in HCC compared with normal controls. We also found significant diagnostic and prognostic values of UBE2C in HCC. Promoter methylation of UBE2C was obviously decreased in HCC and was negatively correlated with UBE2C mRNA expression. 10 miRNAs were predicted to potentially bind to UBE2C. *In vitro* assay and bioinformatic correlation analysis together revealed that hsa-miR-193b-3p might be another key upstream regulatory mechanism of UBE2C in HCC. In conclusion, UBE2C is overexpressed in HCC and may serve as a key diagnostic/prognostic biomarker for patients with HCC.

## INTRODUCTION

Hepatocellular carcinoma (HCC), accounting for 90% of all liver cancer cases, is the sixth most common cancer, and its prevalence is gradually growing all over the world [1, 2]. More than high morbidity, HCC is also an extremely lethal disease, ranking one of the most leading causes of cancer-associated deaths globally [3]. Due to lack of sensitive diagnostic biomarkers, lots of HCC patients are deprived of operative opportunity as they usually present as advanced stage at the fist diagnosis. Though huge advances have been achieved during the past decades, few alternative treatments make available for advanced stage HCC [4]. Moreover, tumor relapse and distant metastasis will finally appear to parts of HCC patients who receiving radical surgery, with the five-year survival rate no more than 12% [5]. Thus, it is a quite significant event to seek and develop potential and effective biomarkers in HCC.

Ubiquitin-conjugating enzyme E2C (UBE2C), encoded by the gene *UbcH10*, belongs to the E2 family [6]. UBE2C functions as a critical regulator of multiple biological processes and its dysregulation appears in many human cancers, including melanoma [7], gastric cancer [8], non-small cell lung cancer [9], rectal cancer [10] and esophageal squamous cell carcinoma [11]. The study conducted by the group of Xiong Y demonstrated that UBE2C functioned as an important oncogene in promoting proliferation, invasion, migration and drug resistance of HCC [3]. Wei et al. also suggested that UBE2C might be a promising therapeutic target for HCC according to GEO and TCGA databases [12]. However, the roles of UBE2C in diagnosing HCC and predicting prognosis of patients with HCC are still unknown. Moreover, the mechanisms causing aberrant regulation of UBE2C in HCC need to be further explored.

In this work, we firstly determined UBE2C expression in HCC by performing integrated analysis of several online databases, then evaluated diagnostic and prognostic values of UBE2C in HCC, and, at the end, explored two mechanisms leading to dysregulation of UBE2C in HCC. These findings from the present study indicate that overexpressed UBE2C, mediated by dysregulated promoter methylation level and suppression of hsa-miR-193b-3p degradation, may serve as a promising diagnostic or prognostic biomarker for HCC.

## MATERIALS AND METHODS

### Oncomine database analysis

Oncomine database [13] was utilized to analyze UBE2C expression in HCC samples compared with normal liver samples using “Meta expression analysis” and “Differential expression analysis” provided by the database. *P*-value < 0.05, Fold change (FC) > 2 and gene rank in the top 10% were set as the thresholds for selecting the datasets of interest.

### GEPIA database analysis

GEPIA (http://gepia.cancer-pku.cn/detail.php) database was employed to further determine UBE2C expression in HCC and normal controls [14]. *P*-value < 0.05 and Fold change (FC) > 2 were considered as statistically significant. Besides, GEPIA database was also introduced to analyze expression difference of UBE2C among various major stage in HCC. *P*-value < 0.05 was considered as statistically significant.

### ROC curve analysis

The expression data of UBE2C from 50 normal liver samples and 374 HCC samples were downloaded from TCGA database. ROC curve analysis was used to assess the diagnostic value of UBE2C in HCC. *P*-value < 0.05 was considered to indicate statistical significance.

### Kaplan-Meier plotter database analysis

Kaplan-Meier plotter (http://kmplot.com/analysis) was used to analyze the prognostic role of UBE2C in HCC as we previously described [15–17]. Four prognostic indices, containing OS, RFS, PFS and DSS, were included. UBE2C was firstly put into the database. Based on the median expression level, a low-group and a high-group were generated. Logrank *P*-value, hazard ratio (HR) and 95% confidence interval (CI) were automatically calculated and presented on the webpage. Logrank *P*-value < 0.05 was considered as statistically significant.

### UALCAN database analysis

UALCAN (http://ualcan.path.uab.edu/index.html) [18] was utilized to detect the promoter methylation level of UBE2C in normal liver samples and HCC samples. *P*-value < 0.05 was considered as significantly different.

### cBioPortal database analysis

cBioPortal (http://cbioportal.org) was employed to assess the relationship between the promoter methylation level of UBE2C and UBE2C mRNA expression level in HCC [19].

### miRNet database analysis

miRNet database (http://www.mirnet.ca/) [20] was introduced to predict potential upstream binding miRNAs of UBE2C, after which a miRNA-UBE2C regulatory network was established using Cytoscape software (Version 3.6.0).

### Cell culture

The human HCC cell lines Huh7 and HepG2 were kindly provided by the First Affiliated Hospital of Medical College, Zhejiang University (Hangzhou, China). Huh7 and HepG2 were cultured in Dulbecco’s Modified Eagle’s Medium (DMEM; Gibco, 12430047) supplemented with 10% fetal bovine serum (FBS; Biological Industries, 04-0101-1, Cromwell, CT, USA) under a humidified atmosphere of 5% CO_2_ at 37°C as previously described.

### Cell transfection

Huh7 and HepG2 cells were firstly seeded into six-well plates, after which miRNA mimics, NC mimics, miRNA inhibitors and NC inhibitors purchased from RiboBio Co., Ltd. (Guangzhou, China) were transfected into these cells at indicated concentrations (50 nM for miRNA mimics and NC mimics; 100 nM for miRNA inhibitors and NC inhibitors) by Lipofectamine™ 3000 (Invitrogen, Shanghai, China) based on the manufacturer’s instruction.

### RNA isolation and qRT-PCR

RNA isolation and qRT-PCR were performed as previously described [21, 22]. The total RNA of HCC cells was extracted by RNAiso plus Reagent (TaKaRa, Kusatsu, Japan), after which total RNA was reversely transcribed into complementary DNA (cDNA) using the PrimeScript RT Reagent Kit (TaKaRa, RR0037A). Subsequently, q-PCR was conducted in triplicates in a Roche LightCycler480 II Real-Time PCR Detection System by SYBR Premix Ex Taq (TaKaRa, RR420A) based on the formula of 2^−ΔΔct^. GAPDH was used as the internal control. UBE2C forward primer sequence: 5′-GGATTTCTGCCTTCCCTGAA-3′; UBE2C reverse primer sequence: 5′-GATAGCAGGGCGTGAGGAAC-3′; GAPDH forward primer sequence: 5′-AATGGACAACTGGTCGTGGAC-3′; GAPDH reverse primer sequence: 5′-CCCTCCAGGGGATCTGTTTG-3′.

### starBase database analysis

starBase database [23, 24] was employed to determine the correlation between UBE2C expression and hsa-miR-193b-3p expression in HCC. *P*-value < 0.05 was considered as statistically significant.

### Statistical analysis

Most of the statistical analyses were conducted by the online tools or databases as mentioned above. Experimental results were presented as mean ± standard deviation (SD) and statistical analysis was conducted using GraphPad Prism software (Version 7). The differences between two groups were assessed using a two-tailed Student’s *t*-test. *P*-value < 0.05 was considered as to indicate a statistically significant difference.

## RESULTS

### UBE2C expression is upregulated in HCC

**Figure 1:**
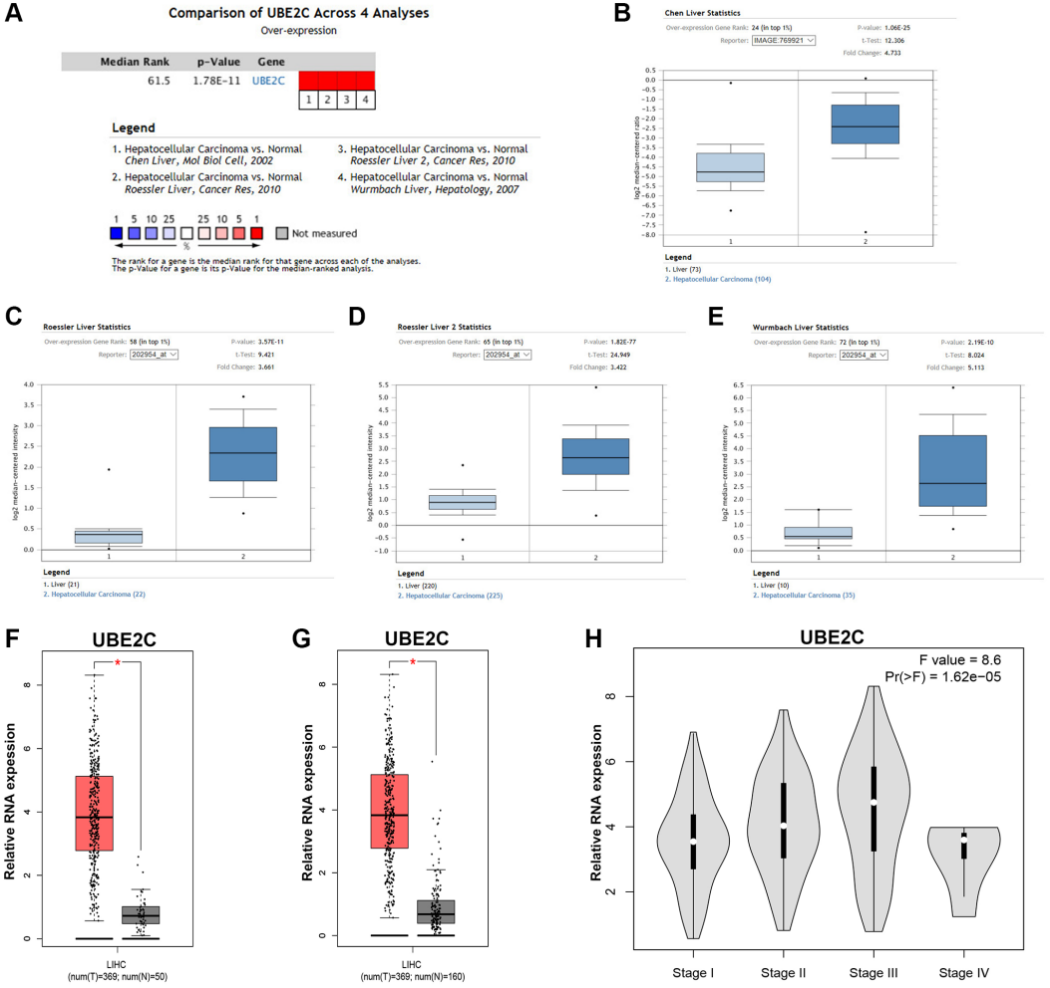
**The expression level of UBE2C in HCC.** (**A**) The details of the meta-analyses of UBE2C in HCC from Oncomine database. The legends below the meta-analytical result present detailed information of the 4 selected datasets. UBE2C expression in patients with HCC based on the Chen Liver dataset (**B**), Roessler Liver dataset (**C**), Roessler Liver 2 dataset (**D**) and Wurmbach Liver dataset (**E**) from Oncomine database. (**F**) UBE2C expression in 369 HCC samples (TCGA) compared with 50 normal controls (TCGA) from GEPIA database. (**G**) UBE2C expression in 369 HCC samples (TCGA) compared with 160 normal controls (TCGA and GTEx) from GEPIA database. (**H**) Expression difference of UBE2C among various major stage of HCC from GEPIA database. “^*^” represents *P*-value < 0.05.

At the first step, we detected UBE2C expression in HCC by comprehensively analyzing several databases. Through using the method of meta-analysis and differential analysis of 4 included datasets in Oncomine database, we found that UBE2C was significantly upregulated in HCC tissues when compared with normal controls, as presented in Figure 1A–1E. Next, GEPIA database was utilized to confirm UBE2C overexpression in HCC. 369 HCC tissue samples and 50 normal liver tissue samples from TCGA database were used. As shown in Figure 1F, UBE2C expression in HCC was markedly higher than that in normal controls. Considering the low count of normal samples, 110 normal liver tissue samples from GTEx database were also included for UBE2C expression analysis. Figure 1G demonstrated that UBE2C expression was significantly increased in HCC, identical with the analytic results from Oncomine database. Expression difference of UBE2C among various stages of HCC was also assessed using TCGA HCC data. A significant expression difference of UBE2C was observed among 4 different HCC stages as shown in Figure 1H, with F-value = 8.6 and *P*-value = 1.62e-05. Notably, Figure 1H revealed that UBE2C expression increased with the rise of cancer stage. Taken together, comprehensive analyses suggest that UBE2C is significantly upregulated in HCC.

### UBE2C serves as a diagnostic and prognostic biomarker for patients with HCC

**Figure 2:**
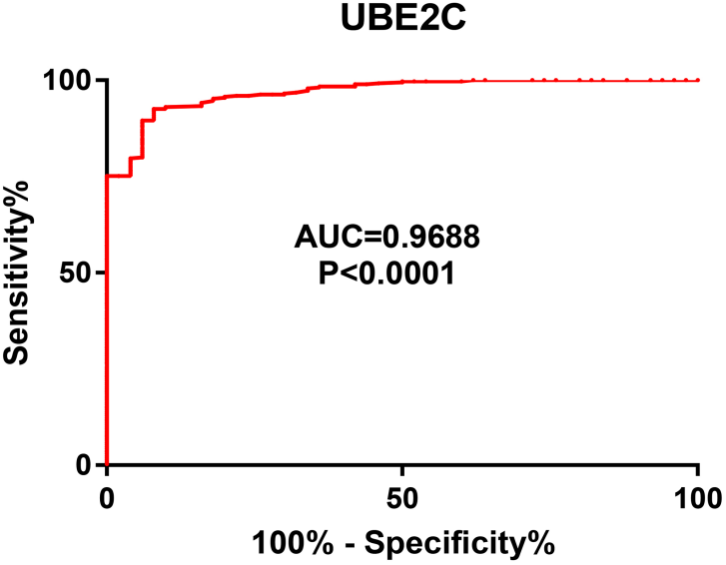
The diagnostic value of UBE2C in HCC.

**Figure 3:**
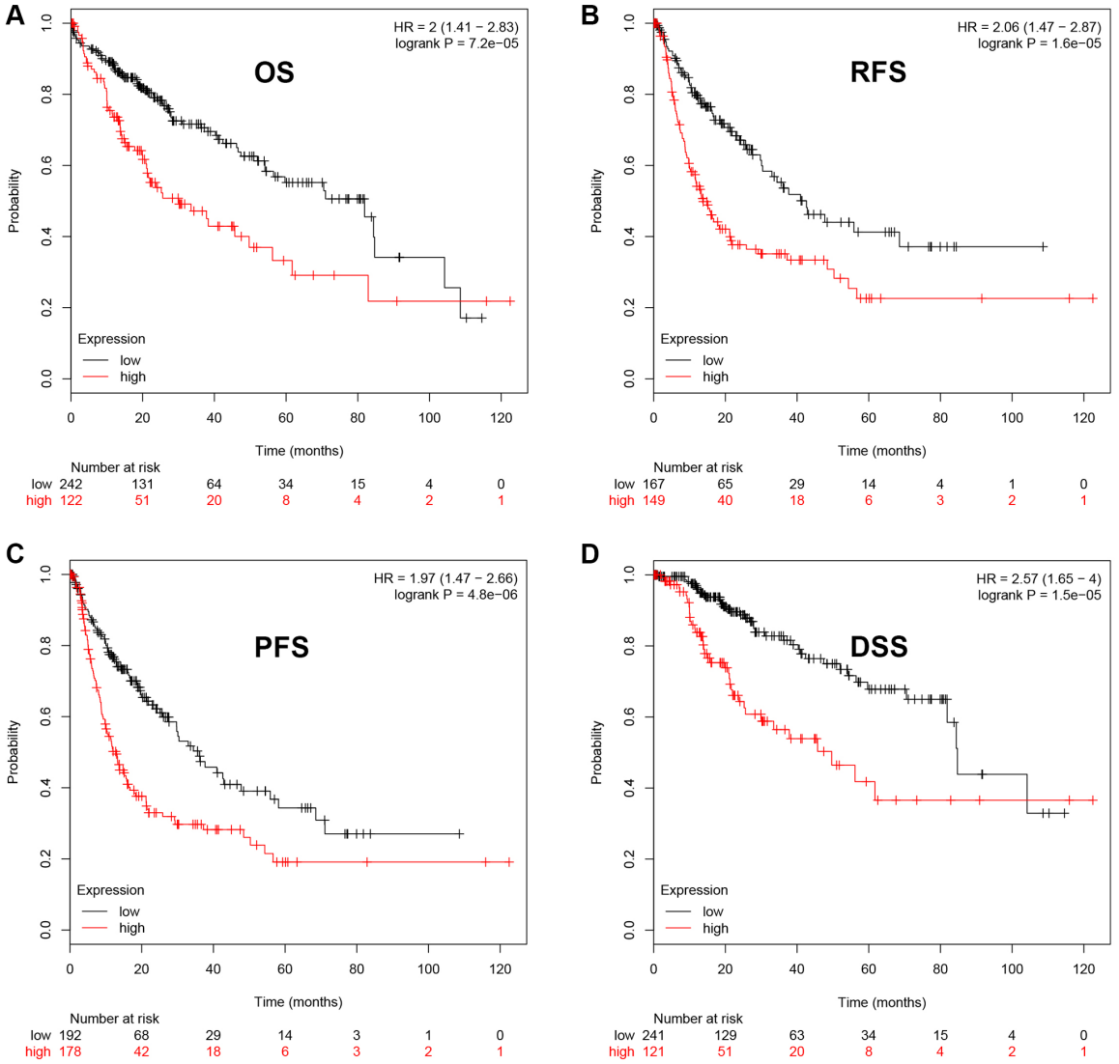
**The prognostic value of UBE2C in HCC.** HCC patients with higher expression of UBE2C indicated a poorer OS (**A**), RFS (**B**), PFS (**C**) and DSS (**D**). Logrank *P*-value < 0.05 was considered as statistically significant. Abbreviations: OS: overall survival; RFS: relapse free survival; PFS: progression free survival; DSS: disease specific survival.

**Figure 4:**
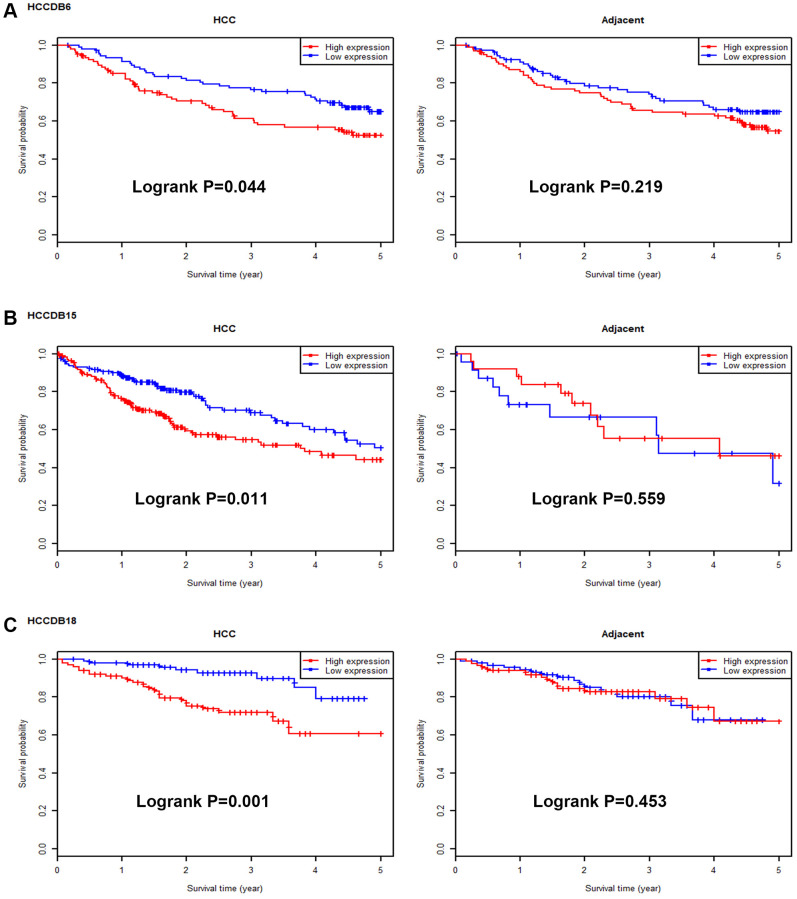
**Survival analysis for UBE2C in HCC and adjacent normal samples.** The prognostic values of UBE2C in HCC and adjacent normal liver tissues in HCCDB6 (**A**), HCCDB15 (**B**) and HCCDB18 (**C**) datasets determined by HCCDB database.

Next, we intended to ascertain if UBE2C possessed diagnostic or prognostic value for patients with HCC. Firstly, TCGA normal and HCC data were introduced to evaluate the diagnostic role of UBE2C in HCC using AUC analysis. As presented in Figure 2, UBE2C had a statistically significant diagnostic value for patients with HCC, with AUC = 0.9688. Subsequently, survival analysis of UBE2C in HCC was also performed using TCGA HCC data by Kaplan-Meier plotter database. In this part, four prognostic indices, containing OS, RFS, PFS and DSS, were included. As shown in Figure 3A, HCC patients with higher expression of UBE2C possessed worse OS (HR = 2.00, 95% CI = 1.41–2.83, logrank *P* = 7.2e-05). Figure 3B suggested that increased expression of UBE2C indicated poor RFS in HCC (HR = 2.06, 95% CI = 1.47–2.87, logrank *P* = 1.6e-05). Upregulation of UBE2C was significantly linked to poor PFS in HCC (HR = 1.97, 95% CI = 1.47–2.66, logrank *P* = 4.8e-05) (Figure 3C). Moreover, high expression of UBE2C was markedly associated with unfavorable DSS of patients with HCC (HR = 2.57, 95% CI = 1.65–4.00, logrank *P* = 1.5e-05) (Figure 3D). As shown in Figure 4A–4C, high expression of UBE2C indicated poor prognosis in HCC but no statistical significance of UBE2C in adjacent normal tissues was observed. All these findings show that UBE2C may serve as a potential diagnostic and prognostic biomarker for patients with HCC.

### Promoter methylation level of UBE2C is decreased in HCC

**Figure 5:**
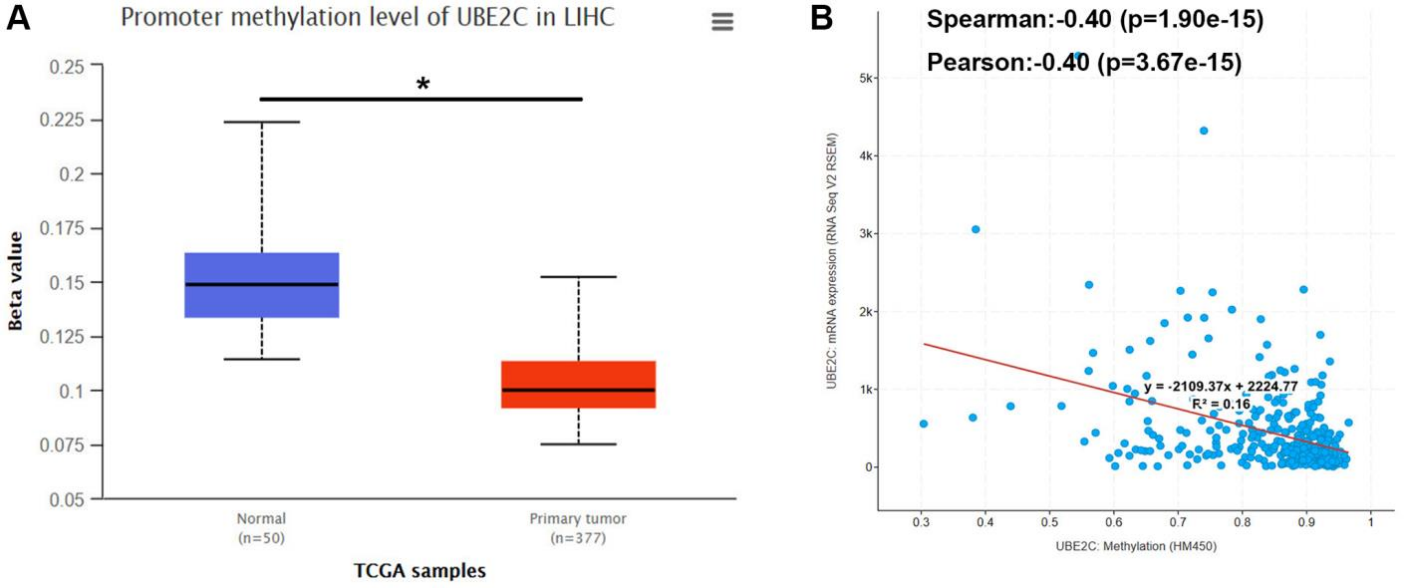
**Promoter methylation level of UBE2C in HCC.** (**A**) UBE2C promoter methylation level was significantly decreased in HCC compared with normal controls determined by UALCAN database. (**B**) UBE2C promoter methylation level was negatively linked to UBE2C mRNA expression determined by cBioPortal database. “^*^” represents *P*-value < 0.05.

It has been widely acknowledged that promoter methylation level of genes may inversely influence gene expression. In view of this regulatory mechanism together with high expression of UBE2C in HCC compared with normal controls, we intended to know if the promoter methylation level of UBE2C was lower in HCC tissue samples than that in normal liver tissues. First of all, we determined the promoter methylation level of UBE2C in normal liver tissue and HCC tissue using TCGA data by UALCAN database. As presented in Figure 5A, the promoter methylation level of UBE2C in HCC was significantly decreased when compared with normal controls. Subsequently, we conducted correlation analysis between promoter methylation level of UBE2C and UBE2C mRNA expression level in TCGA HCC samples using cBioPortal database. Figure 5B demonstrated that UBE2C promoter methylation level was negatively correlated with UBE2C mRNA expression level, with R = −0.4. Altogether, the hypomethylated promoter of UBE2C might account for its high expression in HCC.

### UBE2C expression is negatively regulated by hsa-miR-193b-3p in HCC

**Figure 6:**
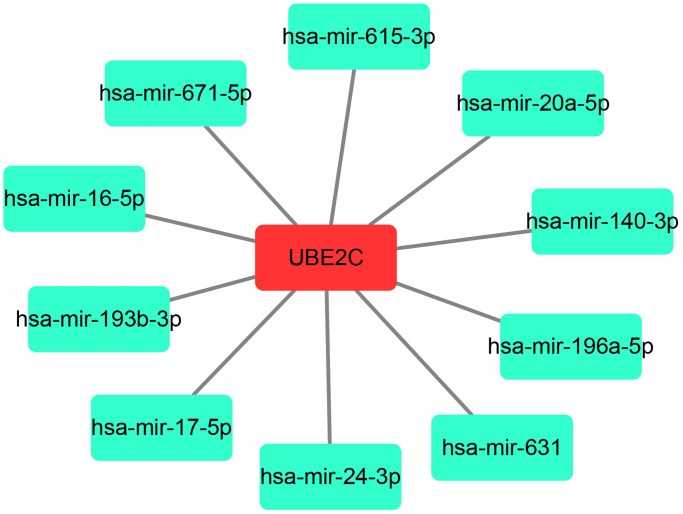
A potential miRNA-UBE2C regulatory network established by Cytoscape software (Version 3.6.0).

**Figure 7:**
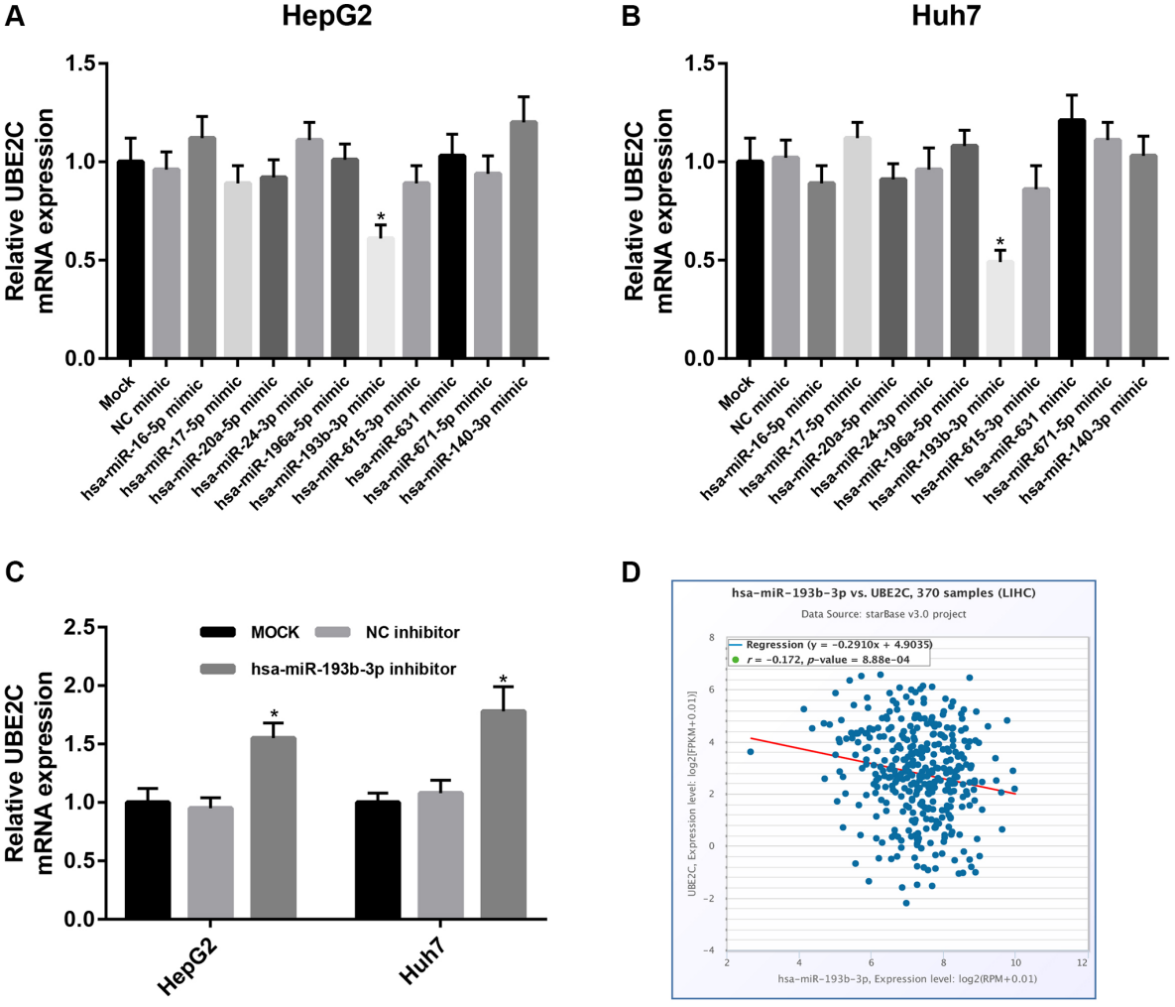
**Identification of hsa-miR-193b-3p as a key regulator of UBE2C in HCC.** Expression change of UBE2C after overexpression of 10 potential upstream miRNAs in HepG2 (**A**) and Huh7 (**B**) cell lines. (**C**) Expression change of UBE2C after silence of 10 potential upstream miRNAs in HepG2 and Huh7 cell lines. (**D**) Hsa-miR-193b-3p expression was significantly negatively correlated with UBE2C expression in HCC determined by starBase database. “^*^” represents *P*-value < 0.05.

As is known to all, gene expression may also be negatively modulated by miRNAs. Therefore, we predicted the potential binding miRNAs of UBE2C through miRNet database, which is an online tool for miRNA-associated studies. Finally, 10 possible miRNAs, including hsa-miR-16-5p, hsa-miR-17-5p, hsa-miR-20a-5p, hsa-miR-24-3p, hsa-miR-196a-5p, hsa-miR-193b-3p, hsa-miR-615-3p, hsa-miR-631, hsa-miR-671-5p and hsa-miR-140-3p, were forecasted. For better visualization, a miRNA-UBE2C regulatory network was constructed as presented in Figure 6. In the next step, we detected the expression change of UBE2C after overexpression of the ten miRNAs in two HCC cell lines, HepG2 and Huh7. Figure 7A showed that UBE2C expression was only negatively regulated by hsa-miR-193b-3p among all the 10 miRNAs in HepG2. As presented in Figure 7B, among all miRNA mimic groups, UBE2C expression was only significantly decreased in hsa-miR-193b-3p mimic group, when compared with NC mimic group. Next, we further assessed the expression change of UBE2C after knockdown of hsa-miR-193b-3p in HepG2 and Huh7 cell lines. The result suggested that UBE2C was significantly upregulated after inhibition of hsa-miR-193b-3p in both HepG2 and Huh7 cell lines (Figure 7C). Moreover, we also found that hsa-miR-193b-3p expression was statistically negatively associated with UBE2C expression in TCGA HCC samples by using starBase database (Figure 7D). These current findings indicate that UBE2C expression is targeted by hsa-miR-193b-3p in HCC.

## DISCUSSION

UBE2C, a member of the E2 enzyme family, plays key roles in ubiquitin-proteasome system. Numerous studies have reported that its dysregulation results in a variety of human cancers, including HCC. However, to the best of our knowledge, the diagnostic and prognostic values of UBE2C in HCC are still not be determined, and its dysregulated mechanisms in HCC are also not be elucidated.

The current study confirmed that UBE2C was significantly upregulated in HCC samples when compared with normal liver samples by the method of comprehensive bioinformatic analysis based on Oncomine and GEPIA databases. Moreover, its expression in advanced stage HCC was markedly higher than that in early-stage HCC. Previous studies also showed that UBE2C exhibited high expression in HCC [3, 12], further supporting analytic accuracy of the present work. Next, we found a significant diagnostic role of UBE2C in HCC according to the TCGA normal liver and HCC data. Subsequently, the survival analysis was also performed to assess the prognostic value of UBE2C in HCC. The results demonstrated that HCC patients with higher expression of UBE2C indicated poorer OS, RFS, PFS and DSS. All these findings show that UBE2C is obviously increased in HCC and its upregulation may act as a significant diagnostic or prognostic biomarker for patients with HCC.

Next, we started to probe the potential mechanisms causing upregulation of UBE2C. Abnormal methylation of promoters could lead to activation of oncogenes or silence of tumor suppressor genes [25, 26]. We intended to ascertain if dysregulation of methylation was involved in UBE2C overexpression in HCC. Therefore, the promoter methylation level of UBE2C in HCC was detected based on the TCGA data using UALCAN database. An evident decrease of UBE2C promoter methylation was observed in HCC compared with normal controls. We also found that UBE2C promoter methylation level was negatively linked to UBE2C mRNA expression in HCC. These current findings suggest that hypomethylation of UBE2C promoter may account for UBE2C overexpression in HCC.

Our team and other labs have well documented that miRNAs are also reported to directly suppress downstream target genes [17, 21, 22, 27–29]. In this study, we also precited the upstream miRNAs of UBE2C using an integrated miRNA study-associated database, namely miRNet. Finally, 10 potential miRNAs (hsa-miR-16-5p, hsa-miR-17-5p, hsa-miR-20a-5p, hsa-miR-24-3p, hsa-miR-196a-5p, hsa-miR-193b-3p, hsa-miR-615-3p, hsa-miR-631, hsa-miR-671-5p and hsa-miR-140-3p) were forecasted. The upstream miRNAs of oncogenic UBE2C should be tumor suppressive miRNAs in HCC. Some of the 10 potential miRNAs have been reported to function as negative regulators in development and progression of HCC. For example, Cheng et al. found that hsa-miR-16-5p inhibited invasion and migration of HCC by targeting IGF1R [30]; Li et al. showed that miR-140-3p enhanced the sensitivity of HCC cells to sorafenib by directly suppressing PXR [31]. Among the 10 potential miRNAs, we found that UBE2C expression was significantly decreased in hsa-miR-193b-3p mimic group whereas UBE2C expression was obviously increased in hsa-miR-193b-3p inhibitor group in HepG2 and Huh7 cell lines. Furthermore, we also found that hsa-miR-193b-3p expression was negatively correlated with UBE2C expression in HCC by starBase database. Additionally, hsa-miR-193b-3p was found to function as a tumor suppressor in the development of HCC [32]. Taken together, hsa-miR-193b-3p may be an upstream modulator of UBE2C in HCC.

**Figure 8:**
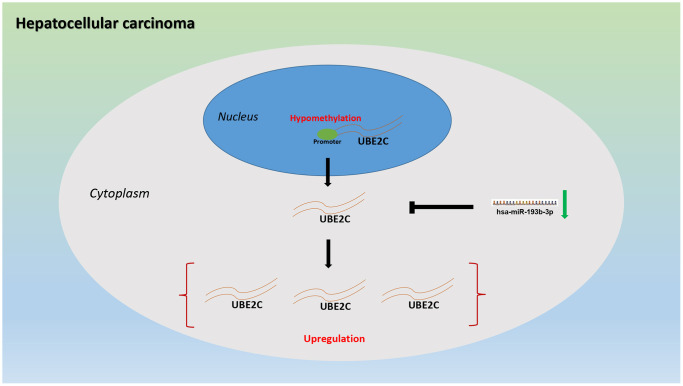
The dysregulated mechanism graph of UBE2C in HCC.

In conclusion, the present study has suggested that UBE2C is significantly upregulated in HCC and a significant diagnostic/prognostic value of UBE2C in HCC is observed. Moreover, we also find two potential dysregulated mechanisms of UBE2C in HCC, including hypomethylated promoter of UBE2C and loss of inhibition of hsa-miR-193b-3p (Figure 8). However, experimental validation should be conduct for these findings in the future.
